# Basolateral Amygdala but Not Medial Prefrontal Cortex Contributes to Chronic Fluoxetine Treatments for PTSD Symptoms in Mice

**DOI:** 10.1155/2020/8875087

**Published:** 2020-11-25

**Authors:** Ying Hao Yu, Chen Yin Ou, Bai Chuang Shyu, Andrew Chih Wei Huang

**Affiliations:** ^1^Department of Psychology, Fo Guang University, Yilan County 26247, Taiwan; ^2^Department of Biotechnology and Animal Science, National Ilan University, Yilan County 26247, Taiwan; ^3^Institute of Biomedical Sciences, Academia Sinica, Taipei, Taiwan

## Abstract

Do chronic fluoxetine treatments reduced footshock-induced posttraumatic stress disorder (PTSD) symptoms, including fear and comorbid depression, in the situational reminder phase? Moreover, are the subareas of the medial prefrontal cortex (mPFC), including the cingulate cortex 1 (Cg1), prelimbic cortex (PrL), infralimbic cortex (IL), and basolateral amygdala (BLA), involved in the fluoxetine amelioration of PTSD symptoms? These two crucial issues were addressed in the present study. All mice were injected with chronic fluoxetine or normal saline treatments for the adaptation (14 days), footshock fear conditioning (1 day), and situational reminder (3 days) phases. After adaptation, the mice were subjected to footshock (2 mA, 10 seconds) or nonfootshock and stayed 2 min in a footshock box for 2 min for fear conditioning. Later, they were placed in the footshock box for 2 min in the situational reminder phase. In the final session of the situational reminder phase, a forced swimming test (FST) and immunohistochemical staining were conducted. The results indicated that footshock induced fear and comorbid depression. Meanwhile, chronic fluoxetine treatments reduced fear and depression behaviors. The Cg1, PrL, IL, and BLA were seemingly to increase c-Fos expression after footshock-induced PTSD symptoms in the situational reminder phase. The fluoxetine treatments reduced only the BLA's c-Fos expression. The findings suggest that BLA contributes to the fluoxetine amelioration of PTSD symptoms; however, the mPFC, including the Cg1, PrL, and IL, did not mediate PTSD symptoms' amelioration stemming from fluoxetine. The present data might help us to further understand the neural mechanism of fluoxetine treatments in PTSD symptoms.

## 1. Introduction

Posttraumatic stress disorder (PTSD) is associated with reexperiencing a traumatic memory, emotional numbering, hyperarousal, the avoidance of cue-associated trauma, and fear and horror [1]. Moreover, PTSD patients suffer from comorbid depression and anxiety symptoms [2, 3]. Previous studies of PTSD involving animal models often examined fear conditioning or fear extinction [4]; however, the present study used the situational reminder procedure with the traumatic memory associated with the context. This kind of study was aimed at mimicking the reexperiencing of a traumatic memory of PTSD symptoms in humans [5, 6].

Fluoxetine is a category of specific serotonin reuptake inhibitor (SSRI) drugs and can effectively ameliorate depressive behavior for patients with major depression disorder [7]. Recently, some studies have shown that fluoxetine might be an effective drug for reducing PTSD's symptoms and for changing its pathological response in the brain [8–11]. For example, PTSD patients who had suffered traumatic events early in life were each given 20-80 mg/day for a continuous period of 8 to 32 weeks, and it decreased the severity of their PTSD symptoms [8]. Microinjections of fluoxetine into the amygdala or hippocampus were found to reduce neurometabolic abnormalities in the amygdala or hippocampus in a single-prolonged, stress-induced PTSD animal model [9]; moreover, another study demonstrated that chronic treatments of fluoxetine could prevent inflammatory gene expression in the anterior cingulate cortex and decrease PTSD symptoms [11]. Fluoxetine administrations in PTSD patients were also shown to recover PTSD-induced synaptic protein loss and dysfunction behaviors [10]. Therefore, SSRI drug fluoxetine is a crucial treatment for PTSD symptoms. This current study examined whether PTSD's fear and comorbid depression behaviors were decreased by fluoxetine treatments, especially in the situational reminder phase of a traumatic memory.

A growing body of evidence has shown that the medial prefrontal cortex (mPFC)-amygdala pathway plays an essential role in regulating PTSD symptoms [12–14]. For example, some recent evidence has suggested that mPFC has an emotional downregulation function to inhibit the negative emotions of the amygdala, whereas the amygdala transfers its property and valence of emotions to the mPFC for the interpretation of emotions [15]. Therefore, the information in mPFC-amygdala connectivity is reciprocal between these two areas [16]. Some studies have demonstrated that the mPFC normally inhibits the activity of the amygdala, resulting in the extinction of fear conditioning [17, 18]. Moreover, the mPFC-amygdala circuitry can be altered by fear-conditioned learning and is involved in the extinction and reinstatement of fear [19]. A neuroimaging study showed that the mPFC-amygdala pathway may govern stress and anxiety disorders [20]. Furthermore, there is an inhibition deficit from the mPFC to the amygdala due to the PTSD symptoms. Moreover, PTSD patients showed hypoactivity of the mPFC and hyperactivity of the amygdala [13].

Recently, many studies have narrowed down the subarea of the mPFC and amygdala, and this line of studies showed that the subareas of the mPFC and amygdala contributed to different functions in the fear conditioning and fear extinction of the PTSD symptoms [4, 21–23]. For example, a review paper reported that the prelimbic cortex (PrL) of mPFC regulates fear expression; meanwhile, the infralimbic cortex (IL) of mPFC controls fear suppression [4]. Moreover, another study demonstrated that the activity of the PrL neurons promotes the extinction of fear conditioning; however, the neuronal activity of the IL inhibits fear behavior after extinction [22]. In addition, BLA, a portion of the amygdala in the basolateral parts, was involved in PTSD symptoms [21, 23]. For example, the high-frequency stimulation of the bilateral BLA was shown to reduce the avoidance behavior in the predator scent-induced PTSD animal model [21]. Furthermore, the transcription factor NF-*κ*B inhibitions of BLA interfered with the amygdala-dependent auditory fear conditioning in the memory retention phase of PTSD [23]. However, no research has systematically examined whether Cg1, PrL, IL, and BLA are involved in fluoxetine treatments for PTSD symptoms. Therefore, this issue was addressed in the present study.

Altogether, the present study addressed the following issues: (a) Do fluoxetine treatments reduce footshock-induced freezing behavior in the situational reminder phase, and do they also ameliorate PTSD's comorbid depression behavior? (b) Are the subareas of the mPFC, including the Cg1, PrL, and IL, and the BLA, involved in PTSD's fear and comorbid depressive behaviors following situational reminders? (c) Do the Cg1, PrL, IL, and BLA contribute to fluoxetine treatments in PTSD's fear and depression symptoms?

## 2. Material and Methods

### 2.1. Animals

Thirty-nine C57BL/6J male mice were bought from the National Laboratory for Animal Breeding and Research Center, Taipei, Taiwan. At the beginning of the experiment, all mice weighed 25-35 grams. The mice were group-housed with another three mice in the plastic cages with wooden bedding. The cage was placed in a colony room with a constant temperature (approximately 23 ± 2°C) and a light phase between 6:00 a.m. and 6:00 p.m. Food and water were provided *ad libitum*. The experiments were performed in compliance with the American Psychological Association ethical standards for the treatment of animals. A description of the details of the treatment was submitted and received approval from a local ethics committee. Every effort was made to minimize the animals' suffering and the number of animals used.

### 2.2. Apparatus

The inescapable footshock apparatus is a box with a surrounding plastic shell measuring 60 cm × 60 cm × 72 cm high. The floor of the apparatus is made of metal grids (0.3 cm diameter at 0.7 cm grid intervals).

### 2.3. Behavioral Procedure

**Figure 1:**
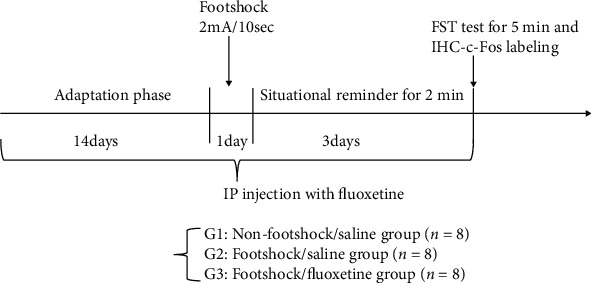
The experimental procedure. After the 14-day adaptation phase, a 2-mA footshock for 10 seconds was applied for fear conditioning, and the freezing time was measured for 2 min during one session a day in the situational reminder sessions. Later, the mice's floating behavior was measured for 5 min in the FST. Following the final session of freezing and floating behavior measurements, 120 mice were sacrificed, and their brain tissues were labeled using immunohistochemical staining with c-Fos proteins.

The procedure of the experiment is shown in Figure 1. After the 14-day adaptation phase, all mice underwent the footshock phase for one day. In this period of time, all mice were intraperitoneally injected normal saline or fluoxetine, placed 2 min in the footshock box, and placed into the nonfootshock/saline, footshock/saline, and footshock/fluoxetine groups (*n* = 8 per group; footshock amount was 2 mA, 10 s). Later, the situational reminder was conducted once a day for three days. All mice were placed 2 min in the same footshock box for situational reminders to reexperience traumatic memories once a day for three days. On the last day of the situational reminders, all mice were also tested using the FST for 5 min. After 120 min of FST testing, all mice were sacrificed, and the brains were removed to label immunohistochemical staining with the c-Fos protein for the specific brain areas. The saline or fluoxetine was continuously administered for the adaptation (14 days), footshock (one day), and situational reminder (three days) phases among the nonfootshock/saline, footshock/saline, and footshock/fluoxetine groups.

### 2.4. Immunohistochemical Staining: c-Fos

Following the last day of the situational reminder test, and 120 min later, all rats were sacrificed via sodium pentobarbital overdose. All mice were perfused with a 0.1-M sodium phosphate-buffered saline buffer (PBS; 100 ml) followed by 4% paraformaldehyde (400 ml) in a 0.1-M PBS buffer. The brain was dissected and postfixed for one day. The tissues of the brain were transferred to 30% sucrose for cryoprotection until the brain tissues sunk to the bottom of the solution. Each whole brain was cut into 40-micrometer coronal sections on a freezing and sliding microtome. All sections of the brain area were then labeled with c-Fos proteins.

For labeling the c-Fos protein, the brain sections were washed once for 15 min in 0.1-M PBS, permeabilized in 3% H_2_O_2_ for 1 h, washed three times in 2% phosphate-buffered saline with Tween 20 (PBST) buffer for 20 min, and soaked in 3% normal goat serum and 1% bovine serum albumin for 1 h. Later, the brain sections were washed twice for 15 min with PBST. Then, the sections were incubated at 4°C overnight with rabbit anti-Fos antibody (Abcam Biotechnology Inc., AB190289, 1 : 1000) for labeling c-Fos. The sections were washed with PBST twice for 15 min and incubated with a biotinylated goat antirabbit secondary antibody (Vector Lab BA-1000, 1 : 500) for 1 h. Later, the sections were washed for 10 min with PBS. The secondary antibody was amplified using the ABC kit (Vector Lab ABC Kit, PK-6100). The positive expression of the brain nucleus was measured via quantification for the selected brain areas. In general, every third section of each brain slice was determined for counting. The c-Fos-positive neurons for each brain session were counted using the software ImageJ. Each group was to be averaged to count the expressions of the c-Fos protein for each brain subarea.

### 2.5. Drugs

Fluoxetine and sodium chloride were purchased from Sigma-Aldrich Company (St. Louis, MO, USA). Sodium chloride was dissolved in distilled water and prepared in 0.9% normal saline. Fluoxetine was dissolved in 0.9% normal saline. Fluoxetine and sodium chloride were administered intraperitoneally. The injection volume of fluoxetine and sodium chloride was 1 ml/kg. The dose of 2.5 mg/kg fluoxetine was used in the behavioral test. The dose of fluoxetine and the continuous fluoxetine injections for 14 days came from the previous study [24].

### 2.6. Statistical Analysis

A 3 × 3 two-way mixed (group vs. session) analysis of variance (ANOVA) was performed for the freezing time among the nonfootshock/saline, footshock/saline, and footshock/fluoxetine groups (*n* = 8 per group). When appropriate, Tukey's honest significant difference post hoc test was conducted. (a) indicated that *p* < 0.05 was considered to be statistically significant between the nonfootshock/saline and footshock/saline groups. (b) indicated that *p* < 0.05 was considered to be statistically significant between the footshock/saline and footshock/fluoxetine groups. One-way ANOVA was conducted to analyze the floating time among the nonfootshock/saline, footshock/saline, and footshock/fluoxetine groups (*n* = 8 per group). When appropriate, Fisher's least significant difference (LSD) post hoc test was conducted. (∗) and (#) indicated that *p* < 0.05 was considered to be statistically significant compared with the nonfootshock/saline and footshock/saline groups, respectively. c-Fos expression numbers were analyzed by one-way ANOVA for the specific brain areas, including Cg1, PrL, IL, BLA, and PC, among the nonfootshock/saline, footshock/saline, and footshock/fluoxetine groups (*n* = 5 per group). When appropriate, Tukey's honest significant difference post hoc test was conducted. (∗) and (#) indicated that *p* < 0.05 was considered to be statistically significant compared with the nonfootshock/saline and footshock/saline groups, respectively.

## 3. Results

### 3.1. Fluoxetine and Freezing Behavior Tests during Situational Reminders

**Figure 2:**
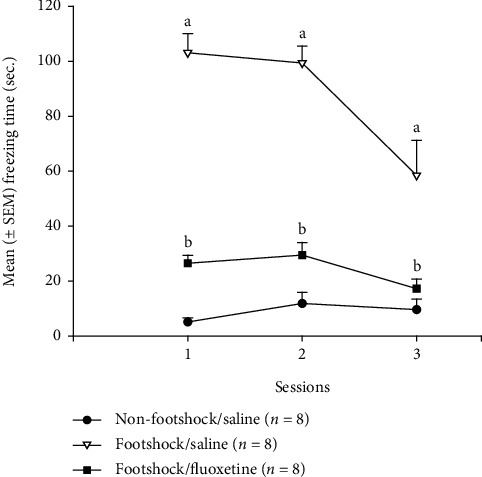
The mean (± SEM) freezing time (sec.) over three sessions of the situational reminder phase for the nonfootshock/saline, footshock/saline, and footshock/fluoxetine groups (*n* = 8, per group).

After encountering severe footshock treatment, mice were placed in the same footshock box, and the freezing time was measured once a day for three days. This was referred to as the situational reminders of traumatic memory. A 3 × 3 mixed two-way ANOVA analysis revealed significant differences in the factors of group (*F* (2, 21) = 73.69, *p* < 0.05), session (*F* (2, 42) = 16.17, *p* < 0.05), and group × session (*F* (4, 42) = 9.39, *p* < 0.05). The post hoc with Tukey test showed that the freezing time of the footshock/saline group was significantly increased compared with the nonfootshock/saline group, indicating that the footshock treatment induced a strong freezing behavior over sessions 1-3 (*p* < 0.05). Moreover, the freezing time of the footshock/fluoxetine group was significantly decreased when compared with the footshock/saline group over sessions 1-3 (*p* < 0.05), indicating the antidepression drug, fluoxetine, and injections could reduce freezing behavior induced by the footshock treatment (Figure 2).

### 3.2. Fluoxetine and PTSD's Comorbid Depression

**Figure 3:**
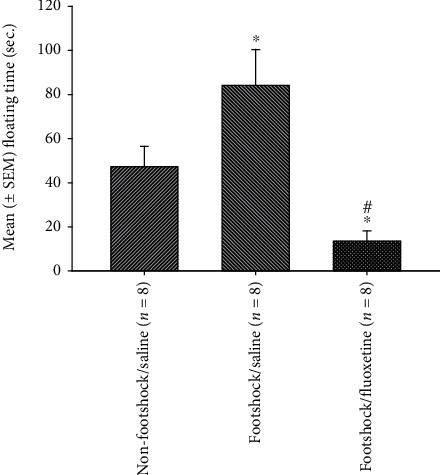
The mean (± SEM) floating time (sec.) in the forced swimming test for the nonfootshock/saline, footshock/saline, and footshock/fluoxetine groups (*n* = 8, per group).

To test the PTSD's comorbid depression symptom of fluoxetine, one-way ANOVA was conducted. The results of the floating time in the FST test showed a significant difference in the factor of group (*F* (2, 21) = 9.86, *p* < 0.05). Furthermore, the post hoc with LSD indicated that the floating time of the footshock/saline group was significantly increased compared with the nonfootshock group (*p* < 0.05). The floating time of the footshock/fluoxetine group was significantly decreased compared with the nonfootshock/saline group (*p* < 0.05). Importantly, the floating time of the footshock/fluoxetine group was significantly decreased compared with the footshock/saline group (*p* < 0.05). In summary, the footshock treatment induced severe depression behavior in the FST test, and the treatment of fluoxetine could reduce the floating behavior. The results mean that injections of antidepression drug fluoxetine reduced PTSD's comorbid depression symptoms (Figure 3).

### 3.3. c-Fos Immunohistochemical Staining and the Amelioration of Fluoxetine in PTSD-Associated Brain Areas

**Figure 4:**
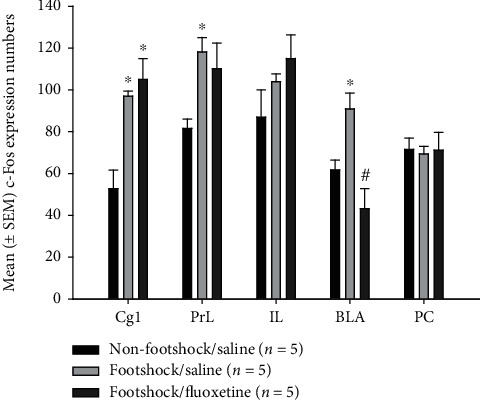
The mean (± SEM) c-Fos-positive neurons per slice in nonfootshock/saline, footshock/saline, and footshock/fluoxetine groups (*n* = 5, per group). The number of c-Fos-positive neurons was counted in the amelioration of fluoxetine for PTSD-associated regions, including the Cg1, PrL, IL, BLA, and PC.

**Figure 5:**
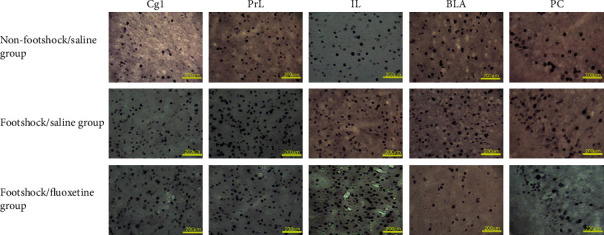
Representative photomicrographs of c-Fos immunoreactivity in the cingulated cortex area 1 (Cg1), prelimbic cortex (PrL), infralimbic cortex (IL), basolateral amygdala (BLA), and piriform cortex (PC) for the nonfootshock/saline, footshock/saline, and footshock/fluoxetine groups (*n* = 5, per group).

For investigating the involvement of brain areas in the amelioration of fluoxetine treatments in PTSD symptoms, a one-way ANOVA analysis was conducted for c-Fos expressions among the nonfootshock/saline group, footshock/saline group, and footshock/fluoxetine group. The results showed that significant differences in the c-Fos expression occurred in the Cg1 (*F* (2, 12) = 12.94, *p* < 0.05), PrL (*F* (2, 12) = 5.18, *p* < 0.05), and BLA (*F* (2, 12) = 10.34, *p* < 0.05). However, there were no significant differences for the c-Fos expression in the IL (*F* (2, 12) = 1.92, *p* > 0.05) and PC (*F* (2, 12) = 0.04, *p* > 0.05). Furthermore, the c-Fos expression of the footshock/saline group was significantly increased compared with the nonfootshock/saline group in the Cg1, PrL, and BLA (*p* < 0.05), indicating that the footshock treatment induced c-Fos expression in the Cg1, PrL, and BLA. The footshock/fluoextine group showed a higher c-Fos expression compared with the nonfootshock/saline group (*p* < 0.05). Importantly, the c-Fos expression of the footshock/fluoxetine group was decreased more than that of the footshock/saline group only in the BLA (*p* < 0.05) but not in the other brain areas. The result of the BLA revealed that the antidepression drug fluoxetine reduced the c-Fos expression induced by footshock (Figures 4 and 5). In conclusion, the results mean that the Cg1, PrL, and BLA were involved in the footshock-induced PTSD symptoms. However, fluoxetine treatments could ameliorate footshock-induced c-Fos expression in the BLA.

## 4. Discussion

In the behavioral tests, footshock treatments induced a long-lasting freezing behavior over three sessions, as mice stayed in the same footshock box and encountered the situational reminders of the traumatic memories. Moreover, the PTSD mice with footshock produced comorbid depression behavior in floating in the FST task. Fluoxetine injections reduced freezing behavior in the situational reminder phase and comorbid depression behavior.

The data of the immunohistochemical staining with c-Fos showed that the subareas of the mPFC, including the Cg1 and PrL but not the IL, were involved in footshock-induced PTSD symptoms, such as freezing and depression behaviors. Only the BLA was shown to be associated with a lower level of c-Fos expression in the footshock/fluoxetine group when compared with the footshock/saline group. The results of the BLA suggest that the fluoxetine treatments ameliorated the negative emotional response.

### 4.1. Comparing the Present Data and the Viewpoint of mPFC-Amygdala Dysfunction in PTSD

How the mPFC-amygdala neural pathway controls PTSD symptoms is an important issue. Based on the hypothesis regarding the mPFC-amygdala dysfunction in PTSD, the medial prefrontal cortex (mPFC) and the amygdala pathway interact with each other to govern emotional processing [25]. The mPFC projects to the amygdala and inhibits the neural activity of the amygdala; thus, the negative emotional effect of the amygdala is distinguished, and healthy people can control their emotional responses [17, 26]. In contrast, the amygdala also projects to the mPFC; thus, the negative emotional information of the amygdala transfers to the mPFC, and the mPFC plays a role in interpreting the valence of emotion from the amygdala for healthy people [17]. In PTSD patients, the neural activity of the amygdala was revealed to be hyperactive when one is reexperiencing a traumatic memory. Meanwhile, the mPFC was shown to be hypoactive; thus, the mPFC cannot inhibit the hyperactivity of the amygdala [27]. Patients with PTSD suffered from negative emotions continuously, and the mPFC-amygdala neural circuit revealed dysfunction [13, 27]. However, the present data were not consistent with this viewpoint regarding the mPFC-amygdala pathway—that the mPFC revealed hypoactivity and the BLA revealed hyperactivity. Instead, the present results showed that the subareas of the mPFC (such as the Cg1, PrL, and IL) and the BLA were associated with hyperactive c-Fos expression, indicating that the mPFC and amygdala exhibited hyperactivity after situational reminders of traumatic memories. This discrepancy in the data might stem from the several reasons outlined below. First, differences in the fear conditioning phase might have resulted in differences in the evidence. The previous studies often tested fear conditioning or fear extinction in the animal model of PTSD; however, the current study manipulated the situational reminder procedure of a traumatic memory. The manipulation of different phases of PTSD may have caused the inconsistent data between the previous studies and our study. Second, the discrepancy data for the previous studies and ours may be due to the differently determined locations of the mPFC and amygdala. The present study determined the location of the Cg1, PrL, and IL for counting the c-Fos expression based on the mouse brain in the seterotaxic coordinates of Paxinos and Franklin [28]. In our study, the range of the Cg1 was AP: +1.77~1.53 mm; ML: +0~0.7 mm; DV: -1~-2 mm. The range of the PrL was AP: +1.77~1.53 mm; ML: +0~0.7 mm; and DV: -1.5~-2.5 mm. The range of the IL was AP: +1.77~1.53 mm; ML: +0~0.7 mm; and DV: -2.5~-3.2 mm [28]. The chosen placements of the brain areas are a bit in the upper part of the brain. Whether the chosen location of the mPFC was due to the discrepancy should be addressed.

In conclusion, the present data on immunohistochemical staining with c-Fos proteins in the mPFC-amygdala neural circuit did not support the hypothesis of hypoactivity in the subareas of the mPFC (i.e., Cg1, PrL, and IL) and hyperactivity in the amygdala. Why this is should be scrutinized in further studies.

### 4.2. Fluoxetine Treatments for PTSD Symptoms

Although the SSRI drug fluoxetine is the first-line medication for treating PTSD symptoms, the effective rate of amelioration in PTSD symptoms is rarely higher than 60%, and less than 20~30% of PTSD patients can obtain full remissions following fluoxetine treatments [29, 30]. Therefore, the therapeutic effect of fluoxetine for PTSD symptoms has some limitations. The animal study showed that following juvenile stress, PTSD animals with chronic fluoxetine treatments at a juvenile age could reduce their PTSD anxiety behavior; however, chronic fluoxetine treatments in adult PTSD animals did not affect PTSD-induced anxiety behaviors. This indicates that the childhood period of time is critical for experiencing a therapeutic effect of fluoxetine for PTSD [31]. Nevertheless, many animal studies on PTSD have demonstrated that fluoxetine treatments can effectively reduce fear-related conditioning and PTSD symptoms. For example, a study on contextual fear conditioning showed that chronic fluoxetine treatments could prevent fear generalization, increase fear extinction, and avoid the occurrence of spontaneous fear recovery [32]. Moreover, previous PTSD animal studies related to the therapeutic effect of fluoxetine found that chronic treatments could decrease sensitized fear behavior [33], reduced hippocampus synaptic proteins [10], and prevented inflammatory responses [11]. Furthermore, the combined treatments of fluoxetine and treadmill exercises have been shown to alleviate PTSD animals' anxiety responses, inhibit the hypothalamus-pituitary-adrenal gland stress system, increase hippocampal brain-derived neurotrophic factor levels, and decrease apoptosis biomarkers. This means that fluoxetine had a therapeutic effect on ameliorating PTSD symptoms and neural and pathology responses [34]. Therefore, despite the fact that some research has suggested that fluoxetine treatments might not be fully effective for curing PTSD symptoms in the clinical setting, fluoxetine is seemingly able to ameliorate fear conditioning and PTSD symptoms in an animal model. Whether fluoxetine treatments can effectively reduce PTSD symptoms should be examined in further studies.

### 4.3. The Involvements of the Neural Substrates in Fluoxetine Treatments for PTSD Symptoms

#### 4.3.1. The mPFC: the Cg1, PrL, and IL in Fluoxetine Amelioration to PTSD

The mPFC has been shown to play different functions, such as emotional regulation, hypothalamic–pituitary–adrenal stress system regulation, working memory, and cognitive execution. Moreover, after repeated stress-related experiences, the dysfunctions of the mPFC [35] and dopamine dysregulation within the mPFC [36] implicated a variety of psychopathologies, such as PTSD, revealing the mPFC's changes in a dendritic spine's density and morphology [35]. Recently, some studies have reported that the subareas of the mPFC-PrL and IL through different neural circuits connected to the subregions of the amygdala control fear expression and fear suppression, respectively [4, 37]. The stress-resilient and susceptible PTSD mice were found to show separated morphological changes in the mPFC; moreover, the stress-resilient mice decreased dendritic numbers in the PrL but increased dendritic numbers in the IL. However, the stress-susceptible mice appeared to only decrease in their dendritic numbers in the IL [38]. However, a little bit of research examined whether the mPFC regulated the fluoxetine amelioration of PTSD [39]. For example, a previous study showed that fluoxetine treatments decreased the freezing behavior and changed the PFC miRNA 1971 expression levels in the animal model of PTSD [39]. The present data showed that although the Cg1 and PrL contributed to the PTSD symptoms, including fear and depression, the Cg1, PrL, and IL were not involved in the PTSD amelioration after chronic fluoxetine treatments. This study might be the first to examine whether the subareas of the mPFC, such as the PrL, IL, and Cg1, contribute to fluoxetine treatments for PTSD using immunohistochemical staining. This issue of the involvement of the mPFC in the fluoxetine amelioration of PTSD symptoms should be scrutinized in further studies.

#### 4.3.2. The Role of BLA in Fluoxetine Treatments of PTSD

Previously, most studies elucidated how BLA contributes to PTSD symptoms. For example, the bilateral stimulation of the amygdala decreased avoidance behavior in predator scent-induced PTSD [21]. Bilateral intra-BLA (but not central amygdala) infusions with sulfasalazine activated the inhibition of NF-*κ*B and disrupted the retention of fear memory in auditory-induced fear conditioning [23]. With deep brain stimulation in the prefrontal cortex, fear and anxiety behaviors were facilitated; however, it decreased the activity of the BLA in the PTSD animal model [40]. Therefore, the BLA might govern fear and anxiety in the PTSD animal model.

On the other hand, fewer studies have examined whether the BLA is involved in fluoxetine treatments for PTSD [9, 11]. Fluoxetine treatments reduced neurometabolites, such as the *N*-acetylaspartate (NAA)/creatine (Cr) and choline moieties (Cho)/Cr ratios, in the amygdala in a single-prolonged stress animal model [9]. Repeated fluoxetine treatments were shown to avoid inflammatory gene expression in the anterior cingulate cortex but not in the BLA [11]. The present data showed that following the experience of footshock, chronic fluoxetine treatments reduced the c-Fos expression in the BLA compared with the group for footshock with saline injections. The findings were not fully consistent with the previous evidence. Whether the BLA was involved in the fluoxetine amelioration of PTSD should be further investigated.

### 4.4. Limitations

Some limitations should be concerned. First, the present study used the typical SSRI antidepressant drugs fluoxetine to ameliorate PTSD symptoms, including freezing and floating behaviors. However, this study did not comprehensively test the other SSRI drugs such as paroxetine or sertraline or the serotonin and norepinephrine reuptake inhibitors (SNRI) such as venlafaxine or duloxetine for the amelioration of PTSD symptoms. It is questioned that SSRIs or SNRIs reduce major depression symptoms; meanwhile, do the other SSRI or SNRI drugs also ameliorate PTSD symptoms, including freezing or floating behaviors? Obviously, this issue should be investigated in further studies. Second, are there any differences of c-Fos expression between the left and right BLA for the amelioration of PTSD symptoms with chronic fluoxetine treatments? In the present study, the left or right parts of the BLA were randomly chosen to count the numbers of c-Fos expression for each brain slice. This study did not, respectively, measure c-Fos expression in the left or right BLA. Thus, the present data of the c-Fos expression in the BLA cannot find a significant difference between the left and right parts of the BLA. This is the second limitation of the present study. This issue has emerged that whether a significant difference for the c-Fos expression occurred in the left and right BLA should be examined further.

### 4.5. Conclusion

Footshock induced fear behavior and comorbid depression in the situational reminder phase of a traumatic memory. However, the fear and comorbid depression were reduced by the chronic treatment of fluoxetine. In immunohistochemical staining data, the Cg1 and PrL (but not IL) of the mPFC, as well as the BLA, contribute to PTSD symptoms in fear and depression behaviors. Importantly, the BLA was involved in the amelioration of fluoxetine treatments in PTSD symptoms, including fear and depression. However, the other brain areas of the mPFC (such as the Cg1, PrL, and IL) did not regulate fluoxetine treatments in the reduction of the PTSD symptoms. This study is the first one that systematically examines the issue of whether the subareas of the mPFC (e.g., Cg1, PrL, and IL) and BLA are involved in fluoxetine treatments for PTSD symptoms. The present data might help us to further understand the neural mechanism of fluoxetine treatments in PTSD symptoms. Furthermore, the present findings should be considered for developing further pharmacological treatments, as these data can offer some clinical implications.

## Data Availability

Data are available from the corresponding author upon reasonable request.
